# Hypoglycaemic and Hypolipidaemic Effects of *Withania somnifera* Root and Leaf Extracts on Alloxan-Induced Diabetic Rats

**DOI:** 10.3390/ijms10052367

**Published:** 2009-05-20

**Authors:** Rajangam Udayakumar, Sampath Kasthurirengan, Thankaraj Salammal Mariashibu, Manoharan Rajesh, Vasudevan Ramesh Anbazhagan, Sei Chang Kim, Andy Ganapathi, Chang Won Choi

**Affiliations:** 1 Department of Biology and Medicinal Sciences, Pai Chai University, Daejeon 302-735, Korea; E-Mails: udayabiochem@yahoo.co.in (R.U.); kimsc@pcu.ac.kr (S.-C.K.); aganapathi2003@rediffmail.com (A.G.); 2 Department of Biotechnology, Bharathidasan University, Tiruchirappalli 620024, Tamilnadu, India; E-Mails: skrrna@gmail.com (S.K.); mariashibu@yahoo.co.in (T.S.M.); rajeshsep25@yahoo.com (M.R.); vrameshanbazhagan@yahoo.com (V.R.)

**Keywords:** alloxan, diabetic, Withania somnifera, hypoglycaemic, hypolipidaemic, flavonoids

## Abstract

*Withania somnifera* is an important medicinal plant, which is used in traditional medicine to cure many diseases. Flavonoids were determined in the extracts of *W. somnifera* root (WSREt) and leaf (WSLEt). The amounts of total flavonoids found in WSREt and WSLEt were 530 and 520 mg/100 g dry weight (DW), respectively. Hypoglycaemic and hypolipidaemic effects of WSREt and WSLEt were also investigated in alloxan-induced diabetic rats. WSREt and WSLEt and the standard drug glibenclamide were orally administered daily to diabetic rats for eight weeks. After the treatment period, urine sugar, blood glucose, haemoglobin (Hb), glycosylated haemoglobin (HbA1C), liver glycogen, serum and tissues lipids, serum and tissues proteins, liver glucose-6-phosphatase (G6P) and serum enzymes like aspartate transaminase (AST), alanine transaminase (ALT), acid phosphatase (ACP) and alkaline phosphatase (ALP) levels were determined. The levels of urine sugar, blood glucose, HbA1C, G6P, AST, ALT, ACP, ALP, serum lipids except high density lipoprotein-bound cholesterol (HDL-c) and tissues like liver, kidney and heart lipids were significantly (p < 0.05) increased, however Hb, total protein, albumin, albumin:globulin (A:G) ratio, tissues protein and glycogen were significantly (p < 0.05) decreased in alloxan-induced diabetic rats. Treatment of the diabetic rats with WSREt, WSLEt and glibenclamide restored the changes of the above parameters to their normal level after eight weeks of treatment, indicating that WSREt and WSLEt possess hypoglycaemic and hypolipidaemic activities in alloxan-induced diabetes mellitus (DM) rats.

## 1. Introduction

Plants have been the major source of drugs for the treatment of diabetes mellitus (DM) in Indian medicine and other ancient systems in the world, and for a long time DM has been treated orally with herbal medicines or their extracts [1], because plant products are frequently considered to be less toxic and more free from side effects than synthetic ones [2]. Furthermore, after the recommendations made by the WHO on DM, investigations on hypoglycaemic agents from medicinal plants have become more important and the search for more effective and safer hypoglycaemic agents has continued to be an important area of active research. World ethnobotanical information about medicinal plants reports that almost 800 plants could be used to control DM. Many herbs and plants have been described as possessing hypoglycaemic activity when taken orally [1,3]. Some of these plants have also been pharmacologically tested and shown to be of some value in human diabetes treatment.

*Withania somnifera* (L.) Dunal, commonly known in Sanskrit as Ashwagandha, is a perennial plant belonging to the order Solanaceae. The pharmacological effects of the roots of *W. somnifera* are attributed to the presence of withanolides, a group of steroidal lactones [4]. Its leaves are used in Ayurvedic and Unani systems for treatment of tumors and tubercular glands [5]. A number of withanolide steroidal lactones have been isolated from the leaves of *W. somnifera* [6] and exhibit antibacterial, anti-fungal and antitumor properties [7]. There are a number of reports elucidating the chemical and pharmacological properties of *W. somnifera* [8,9]. Hypoglycaemic activity of Trasina (an ayurvedic formulation) consisting of *W. somnifera* as one of the important constituents has been established beyond doubt and this activity may be due to its antioxidant properties [10]. It has been in used for a very long time for all age groups and for both sexes and even during pregnancy without any side effects. Tripathy *et al.* [11] have reviewed the traditional uses and antidiabetic activities of *W. somnifera*. Hypoglycaemic effects [12] and the effects of *W. somnifera* on insulin sensitivity in non-insulin dependent DM rats [13] have been reported. The chemistry and nutritional properties of phenolic compounds, including flavonoids, have been extensively reviewed [14]. Flavonoids are commonly found in all plants and also possess hypoglycemic and antidiabetic activities [15]. There is no report on flavonoid-containing extracts of *W. somnifera* relating to antidiabetic and antihyper-lipidaemic activities. Therefore, the present study was aimed at determining the hypoglycaemic and hypolipidaemic effects of *W. somnifera* root (WSREt) and leaf (WSLEt) extracts on alloxan-induced DM.

## 2. Results and Discussion

### 2.1. Alloxan-induced DM

Alloxan has been observed to cause a massive reduction of the β-cells of the islets of Langerhans and induce hyperglycaemic [16]. An observation in this study correlates with the previous research findings, in that the blood glucose level was significantly (p < 0.05) elevated in alloxan-induced diabetic rats [17].

### 2.2. Mechanism of action of the standard drug glibenclamide

Binding of glibenclamide with its receptor leads to the closure of the potassium channels which opens calcium channels for influx of Ca^2+^ ions into the cytoplasm and release of insulin from the pancreatic islets. These K^+^ channels are responsive to ATP/ADP ratio and close when the ratio increases because of an increase in glucose metabolism [18,19]. With chronic glibenclamide treatment, insulin production is not increased and may return to pretreatment values, but insulin efficacy continues and is thought to involve extrapancreatic mechanisms to increase insulin sensitivity in target tissues, such as liver and muscle as well as in other cells like monocytes and erythrocytes. This also leads to decrease hepatic glycogenolysis, gluconeogenesis and blood-glucose concentrations.

### 2.3. Quantification of total flavonoids in WSREt and WSLEt

Several researchers have reported that flavonoids have hypoglycaemic, hypolipidaemic and hypocholesterolaemic effects [20,21]. In the present study, the amount of total flavonoids was determined in WSREt and WSLEt using spectrophotometric analysis (Table 1). The amount of total flavonoids in quercetin equivalents (QE) was in 530 mg/100 g dry weight (DW) in WSREt and 520 mg/100 g DW in WSLEt. From these results, it can be assumed that the flavonoids might be responsible for the hypoglycaemic and hypolipidaemic effects of *W. somnifera* root and leaf extracts.

### 2.4. Effects of WSREt and WSLEt on body weight (bw)

Changes in initial and final bw of normal control and experimental groups are shown in Table 2. Marked bw loss was observed in diabetic rats. The data obtained from this study showed that the treatment of WSREt, WSLEt and glibenclamide protects the diabetic rats from massive bw loss, when given orally, daily for eight weeks. WSREt-, WSLEt- and glibenclamide-treated rats showed a recovery in final bw which was close to that of normal control rats. Moreover, the weight gain was lesser in the diabetic rats when compared to normal control rats. Thus, the bw loss due to catabolic effects seen in diabetic rats was only partially attenuated by the plant extracts.

### 2.5. Effects of WSREt and WSLEt on urine sugar, blood glucose, haemoglobin (Hb), glycosylated haemoglobin (HbA1C) and liver glycogen

Induction of diabetes in the experimental rats was confirmed by the presence of a high blood glucose and low level of liver glycogen. The levels of urine sugar, blood glucose (308%), HbA1C (230%) were significantly (p < 0.05) increased, however Hb (66%) and liver glycogen (58%) were significantly (p < 0.05) decreased in alloxan induced diabetic rats when compared with those of normal control rats. Administration of WSREt and WSLEt at doses of 100 and 200 mg/kg bw to diabetic rats tends to bring the values to near normal. Among these two doses of WSREt and WSLEt, the dose of 200 mg/kg bw showed better results (Table 3). The levels of blood glucose (35%) and HbA1C (45%) were significantly (p < 0.05) decreased, however Hb (143%) and liver glycogen (150%) were significantly (p < 0.05) increased in diabetic rats treated with a dose of 200 mg/kg bw of WSREt when compared with those of diabetic rats. Therefore, the dose of 200 mg/kg bw of WSREt and WSLEt were selected for further analysis of lipid profile, protein profile, important serum and liver enzymes (Tables 4 – 8).

It is not clear how WSREt and WSLEt cure diabetic rats under hyperglycaemic conditions. We assumed that WSREt and WSLEt may increase the pancreatic secretion of insulin from the cells of islets of Langerhan’s or both extracts may act like insulin substitutes. The effect of WSREt and WSLEt on the level of blood glucose in diabetic rats was more pronounced than that of glibenclamide. Moreover, the excess of glucose present in circulation during DM reacts with Hb to form HbA1C, a glycosylated Hb. HbA1C was found to increase in patients with DM and this increase is directly proportional to the fasting blood glucose level [22]. These findings correlate with the present study, in which a significant change was observed in total Hb content. The HbA1C was also significantly (p < 0.05) increased in diabetic rats when compared to that of normal control rats, which indicates that the Hb gets glycosylated in the presence of hyperglycaemia. This is the first study in which a follow-up was made on the changes in HbA1C concentration after the administration of WSREt and WSLEt alone. HbA1C has been found to be high over a long period of time in diabetes [23]. Therefore, the measurement of HbA1C is supposed to be a very vital index for glycaemic control. Glycogen synthesis in the rat liver and skeletal muscles was also impaired during diabetes [24]. Similar result was observed in this study. Diabetic rats treated with WSREt and WSLEt enhanced the rate of glycogenesis as indicated by higher amounts of hepatic glycogen.

### 2.6. Effects of WSREt and WSLEt on serum and tissue lipids profile like total cholesterol (TC), triglycerides (TG) and phospholipids (PL)

Effect of administering WSREt and WSLEt to diabetic rats on serum and tissues lipids like TC, TG, PL, serum high density lipoprotein-bound cholesterol (HDL-c), very low density lipoprotein-bound cholesterol (VLDL-c) and low-density lipoprotein-bound cholesterol (LDL-c) are presented in Tables 4 – 6. The rise in blood sugar is accompanied by the increase in TC, TG, PL, LDL-c, VLDL-c and fall of HDL-c in diabetic rats. The levels of serum TC (201%), TG (147%), PL (161%), VLDL-c (147%) and LDL-c (195%) were significantly (p < 0.05) increased in diabetic rats when compared to those of normal control rats, while the level of serum HDL-c (70%) was significantly (p < 0.05) decreased in diabetic rats when compared to that of normal control rats (Table 4). The serum lipids like TC (54%), TG (75%), PL (65%), VLDL-c (75%) and LDL-c (64%) were significantly (p < 0.05) decreased and HDL-c (132%) was significantly (p < 0.05) increased in diabetic rats treated with WSREt when compared to those of diabetic rats.

The abnormal high concentration of serum lipids is mainly due to increase in the mobilization of free fatty acids from the peripheral fat deposits, because insulin inhibits the hormone sensitive lipase production. However, administering WSREt and WSLEt to diabetic rats tends to bring the values to near normal. Thus, WSREt and WSLEt treatments exhibited hypocholesterolaemic, hypotriglyceridaemic and hypophospholipidaemic effects while at the same time increasing the HDL-c.

Furthermore, there were significant increases of TC (163% in liver, 160% in kidney and 212% in heart), TG (150% in liver, 187% in kidney and 255% in heart) and PL (186% in liver, 195% in kidney and 199% in heart) in alloxan induced diabetic rats when compared to those of normal control rats (Tables 5 and 6). On the other hand, TC (65% in liver, 68% in kidney and 52% in heart), TG (70% in liver, 58% in kidney and 45% in heart) and PL (55% in liver, 54% in kidney and 55% in heart) levels were significantly (p < 0.05) decreased in diabetic rats treated with WSREt when compared to those of diabetic rats. As a result, we found that the heart is the most targeted organ for the lipid accumulation in alloxan induced diabetic rats.

Therefore, the elevated level of serum lipids in DM causes the risk of coronary heart disease [25]. It has been well established that DM alters the normal metabolism of tissues like liver, kidney and heart. Like diabetic rats treated with glibenclamide, the administration of WSREt and WSLEt to diabetic rats tends to bring the values to near normal. *W. somnifera* is known to have antioxidant properties [26] and this may reduce the susceptibility of lipids to oxidation and stabilize the membrane lipids thereby reducing oxidative stress.

### 2.7. Effects of WSREt and WSLEt on serum and tissues protein

The levels of total protein in serum (78%), liver (51%), kidney (70%) and heart (67%) and albumin (65%) and albumin:globulin (A:G) ratio (65%) in serum were significantly (p < 0.05) decreased in diabetic rats when compared to those of normal control rats. On the other hand, no change was observed in the level of serum globulin in diabetic rats when compared to that of normal control rats (Table 7). The total protein levels in serum (124%), liver (189%), kidney (141%) and heart (149%) and albumin (147%) and A:G ratio (161%) in serum were significantly (p < 0.05) increased in diabetic rats treated with WSREt when compared to those of diabetic rats. These results are accordance with the glibenclamide treated rats.

Hypoalbuminemia was observed in DM, which is consisted with other report in that the altered A:G ratio was observed in diabetic rats [27]. Distinct metabolic renal alterations lead to a negative nitrogen balance, enhanced proteolysis and lowered protein synthesis in experimental diabetes [28]. The reversal of the changes by WSREt and WSLEt therapy may be proved that the insulin deficiency had been sufficiently corrected. Serum albumin and A:G ratio as well as total protein never deviated from the normal range throughout the treatment period in WSREt and WSLEt treated diabetic rats. *W. somnifera* has been reported to produce anabolic effects, enhancing the synthesis of certain modulator proteins in rat liver and increasing the bw in humans [29].

### 2.8. Effects of WSREt and WSLEt on liver glucose-6-phosphatase (G6P) and serum enzymes like aspartate transaminase (AST), alanine transaminase (ALT), acid phosphatase (ACP) and alkaline phosphatase (ALP) activities

The effects of administering WSREt and WSLEt to diabetic rats on liver G6P and serum enzymes AST, ALT, ACP and ALP are given in Table 8. Alloxan administration increased liver function biomarkers such as ALP and ALT significantly in comparison with normal control rats [30]. The activities of liver G6P (220%) and serum enzymes like AST (181%), ALT (208%), ACP (156%) and ALP (124%) were significantly (p < 0.05) increased in the diabetic rats when compared to those of normal control rats. But the activities of liver G6P (55%) and serum AST (55%), ALT (60%), ACP (71%) and ALP (84%) were significantly (p < 0.05) decreased in diabetic rats treated with WSREt at the dose of 200 mg/kg bw when compared to those of diabetic rats. In addition, the administration of WSLEt at the dose of 200 mg/kg bw also significantly reduced the liver G6P (52%) and AST (71%), ALT (55%), ACP (68%), and ALP (85%) activities in serum of diabetic rats.

In the present study, the key gluconeogenic enzyme G6P activity was assayed in liver of diabetic rats because the liver is the main organ responsible for maintaining homeostasis of blood glucose. The activity of G6P is enhanced during diabetes [31]. The dearrangement in carbohydrate metabolism results in impaired glucose homeostasis leading to hyperglycaemia. WSREt and WSLEt replenished liver glycogen stores and suppressed the hepatic gluconeogenesis by decreasing activity of G6P. This result is accordance with the report of Bhavapriya *et al.* [32], in that the increased G6P activity is reversed by Aavirai kudineer (a herbal formulation) in diabetic rats.

In the present study the activities of AST, ALT, ACP and ALP in serum were altered in DM. In diabetic animals, the changes in the levels of AST, ALT, ACP and ALP are directly related to changes in metabolism in which the enzymes are involved. The increased activities of transaminases, which are active in the absence of insulin due to the availability of amino acids in the blood of DM [30,33] and are also responsible for the increased gluconeogenesis and ketogenesis. The restoration of AST and ALT to their respective normal level was observed in the WSREt and WSLEt treated groups. This is consistent with our previous report of the extracts of *Chinese juniper* berries [17]. AST and ALT levels also act as an indicator of liver function hence restoration of normal level of these enzymes indicates that the normal functioning of liver. Increased activities of serum ACP and ALP have been observed in alloxan diabetic rats [34]. Alloxan treated diabetes caused lipid peroxide mediated tissue damage in the pancrease, liver, kidney, and heart [34]. The increase in the levels of these enzymes in diabetes may be as a result of the leaking out from the tissues and then migrating into the blood stream [31]. Diabetes and hyperlipidaemia also cause cell damage by altering the cell membrane architecture, which results in enhanced activities of ACP and ALP in diabetic rats. In WSREt, WSLEt and glibenclamide treated groups, the cell damage might be reverted and which may leads to the decreased activities of ACP and ALP. Therefore, the present study clearly indicates that WSREt and WSLEt possess hypoglycaemic and hypolipidaemic activities in alloxan induced DM rats.

## 3. Experimental Section

### 3.1. Chemicals

Alloxan and quercetin dihydrate were purchased from Sigma Chemicals Co. (St Louis, Mo, USA) and Aldrich Chemicals Co. (Steineheim, Germany). Cholesterol, 1-amino-2-naphthol-4-sulphonic acid (ANSA), ammonium molybdate, sodium metaperiodate, citric acid monohydrate and acetyl acetone were purchased from Ranbaxy Chemicals (P) Ltd (Mumbai, India). Glycerol trioleate was purchased from Sisco Research Laboratories (P) Ltd (Mumbai, India). l-Aspartic acid, α-ketoglutaric acid, 2,4-dinitrophenyl hydrazine, d-l-alanine and sodium β-glycerophosphate were obtained from Sd. Fine Chemicals, Mumbai, India. Sodium tungstate was obtained from Hi-Media Laboratories (New Delhi, India). All other chemicals and reagents, used in the present study, were of analytical reagent grade with high purity and were purchased from Loba Chemie (P) Ltd and E-Merck Chemicals (P) Ltd., (Mumbai, India) and Nice Chemicals (Kerala, India).

### 3.2. Animals

The experiments were carried out in forty two male healthy adult Albino Wistar strain rats (150 – 180 g bw) procured from an authorized firm in Bangalore, India. They were housed in plastic cages with filter tops under controlled conditions of 12 h light/12 h dark cycle, 50% humidity and 28 ± 2 °C. They all received a standard pellet diet (Sri Saidurga Feed and Foods, Bangalore, India) and water *ad libitum*. The standard pellet diet contained 21% crude protein, 5% fat, 4% crude fiber, 8% ash, 1% calcium, 0.6% phosphorus, 2% vitamins and 55% nitrogen free extract (carbohydrates). The animals were maintained as per the principles and guidelines of the ethical committee for animal care of the Bharathidasan University (Tamilnadu, India) in accordance with the Indian National Law on Animal Care and Use (CPCSEA).

### 3.3. Plant material

Seeds of *W. somnifera* (L.) were procured from the Central Institute of Medicinal and Aromatic Plants (CIMAP), Lucknow, India and the plants were grown in the experimental garden. The plants were collected in the month of June 2006 and identified by Dr. A. Ganapathi, Professor and Botanist, Department of Biotechnology, Bharathidasan University, Tiruchirappalli, Tamilnadu, India. The plant parts like root and leaf were selected for the analysis of total flavonoids and antidiabetic activity.

### 3.4. Preparation of WSREt and WSLEt

The parts (root and leaf) of the plant were collected from field grown plants in six months after planting. The collected parts of medicinal plant were brought into the laboratory, cleaned and dried in shade, and then ground to a fine powder. About 500 g of dry powder was extracted with ethanol (80%) at 70 °C by continuous hot percolation using a Soxhlet apparatus. The extraction was continued for 24 h. The ethanolic extract was then filtered and kept in oven at 40 °C for 24 h to evaporate the ethanol from it. The concentrated extract was then dissolved in as little water as possible and washed three times with chloroform. The residual layer was extracted three times with ethyl acetate. All the extracts were finally pooled and concentrated using the rotary evaporator. A dark brown residue (extract) was obtained. The yield of extracts from root and leaf was about 76 and 72 g, respectively. The extracts were kept separately in air tight containers in a deep freezer until the time of use.

### 3.5. Determination of total flavonoids in WSREt and WSLEt

The content of flavonoids in the extracts of *W. somnifera* root and leaf was determined by spectrophotometrically according to the method of Quettier-Deleu *et al.* [35] using quercetin as a reference compound. One mL of plant extract in methanol (10 mg/mL) was mixed with 1 mL aluminium trichloride in ethanol (20 g/L) and diluted with ethanol to 25 mL. The absorption at 415 nm was read after 40 min at 20 *°*C. Blank sample was prepared from 1 mL plant extract and 1 drop acetic acid, and diluted to 25 mL. The absorption of quercetin solutions was measured under the same conditions. Standard quercetin solution was prepared from 0.05 g/L of quercetin. All determinations were carried out in triplicate. The amount of flavonoids was expressed as QE mg per 100 g of DW.

### 3.6. Induction of diabetes mellitus in rats

The rats were injected with alloxan monohydrate dissolved in sterile normal saline at a dose of 150 mg/kg bw, intraperitoneally. The rats were then kept for the next 24 h on 5% glucose solution bottles in their cages to prevent hypoglycaemia [36]. After a fortnight, rats with moderate diabetes having glycosuria (indicated by Benedict’s test for urine) and hyperglycaemia with blood glucose range of 250 – 300 mg/dL were used for the experiment. Blood was collected from the eyes (venous pool) by sino-ocular puncture for the estimation of the blood glucose.

### 3.7. Experimental design and treatment

In the experiment a total number of 42 rats (36 diabetic induced rats and six normal rats) were used. Diabetes was induced in rats two weeks before starting the treatment. The rats were divided into seven groups as follows after the induction of alloxan diabetes and each group comprised of six rats. Group 1: Normal control rats received only distilled water during the experimental period. Group 2: Diabetic control- freshly prepared alloxan in normal saline was administered in a single dose of 150 mg/kg bw through intraperitoneally to overnight fasted rats and the animals were allowed to develop diabetes for two weeks. Group 3: Diabetic rats were daily treated with WSREt at a dose of 100 mg/kg bw dissolved in distilled water using an intragastric tube for eight weeks. Group 4: Diabetic rats were daily treated with WSREt at a dose of 200 mg/kg bw dissolved in distilled water using an intragastric tube for eight weeks. Group 5: Diabetic rats were daily treated with WSLEt at a dose of 100 mg/kg bw dissolved in distilled water using an intragastric tube for eight weeks. Group 6: Diabetic rats were daily treated with WSLEt at a dose of 200 mg/kg bw dissolved in distilled water using an intragastric tube for eight weeks. Group 7: Diabetic rats were orally treated with glibenclamide (0.6 mg/kg bw) dissolved in distilled water daily for eight weeks.

### 3.8. Collection of samples

The rats were carefully monitored every day and weighed every week. The sugar levels of urine and blood of all the rats were determined. After eight weeks of treatment the rats were sacrificed by cervical dislocation. Blood was collected and processed for the biochemical estimations. Tissues such as liver, kidney and heart were dissected out, washed in ice-cold saline patted dry and weighed accurately and used for lipid extraction by the method of Folch *et al.* [37] and further analysis.

#### 3.8.1. Determination of urine sugar, blood sugar, Hb, HbA1C and liver glycogen

The urine sugar was detected by Benedict’s method [38]. Fasting blood glucose was estimated by the *o*-toluidene method [39]. Hb was estimated by Dacie and Lewis method [40] and HbA1C by Bannon’s method [41]. Liver glycogen was estimated by the method of Carroll *et al.* [42].

#### 3.8.2. Determination of serum lipid profile and tissues lipids like TC, TG and PL

Serum lipids like TC and TG were determined by the method of Zlatkis *et al.* [43] and Foster and Dunn, [44] respectively. PL was estimated by the method of Zilversmit and Davis [45]. Lipoproteins were estimated by the method of Burstein *et al.* [46]. Tissues lipids like TC, TG and PL were also estimated by the above mentioned methods.

#### 3.8.3. Determination of serum and tissues protein

Serum total proteins and tissues proteins from liver, kidney and heart were determined by the method of Lowry *et al.* [47]. Serum albumin was determined by the method of Reinhold [48] and then serum globulin was determined by the following formula: Globulin = total protein – albumin.

#### 3.8.4. Determination of assay of liver G6P and serum enzymes like AST, ALT, ACP and ALP

Activity of G6P in the liver was assayed by the method of Koide and Oda [49]. The activities of serum AST and ALT were assayed by the method of Reitman and Frankel [50]. ACP and ALP were assayed by the method of King [51].

### 3.9. Statistical analysis

Statistical evaluation was done using One Way Analysis of Variance (ANOVA) followed by Duncan’s Multiple Range Test (DMRT) by using SPSS 11.09 for windows. The significance level was set at p < 0.05.

## 4. Conclusions

From the above results, it may be concluded that the *W. somnifera* root and leaf extracts possess antidiabetic and antihyperlipidaemic activities in alloxan-induced diabetic rats. *W. somnifera* root extract contained more flavonoids than leaf extract. Further, phytochemical characterization of *W. somnifera* is required to identify the specific compound(s) involved in the observed hypoglycaemic and hypolipidaemic activities.

## Figures and Tables

**Table 1.** Total content of flavonoids of *Withania somnifera* root and leaf extracts.

**Extracts**	**Flavonoids (mg/100 g dry weight in quercetin equivalents)**
WSREt	530 ± 80
WSLEt	520 ± 60

Values are expressed as mean ± SD of three determinations.

**Table 2.** Effects of *W. somnifera* (L.) extracts on body weight.

**Groups**	**Initial weight (g)**	**Final weight (g)**	**Change in body weight (g)**	**Change in body weight (%)**
Normal control	160.00 ± 8.91	205.17 ± 9.06	45.17 ± 6.78	+ 28.23
Diabetic control	172.00 ± 8.42	152.83 ± 8.78	19.17 ± 5.21	−11.15
Diabetic + WSREt (100 mg/kg body weight)	168.00 ± 9.24	208.00 ± 10.23	40.00 ± 7.64	+ 23.81
Diabetic + WSREt (200 mg/kg body weight)	165.17 ± 10.19	207.33 ± 8.42	42.17 ± 6.42	+ 25.53
Diabetic + WSLEt (100 mg/kg body weight)	165.00 ± 10.37	195.00 ± 7.32	30.00 ± 6.11	+ 18.18
Diabetic + WSLEt (200 mg/kg body weight)	170.00 ± 8.16	212.67 ± 9.64	42.67 ± 7.56	+ 25.10
Diabetic + glibenclamide	170.00 ± 7.89	208.50 ± 8.16	38.50 ± 5.98	+ 22.65

Values are expressed as mean ± SD of six samples from each group. WSREt: *W. somnifera* root extract, WSLEt: *W. somnifera* leaf extract.

**Table 3.** Effects of *W. somnifera* (L.) extracts on urine sugar, blood glucose, haemoglobin, glycosylated haemoglobin and liver glycogen.

**Groups**	**Urine Sugar**	**Blood glucose (mg/dL)**	**Hb (g/dL)**	**HbA1C (mg/gHb)**	**Liver glycogen (mg/g tissue)**
Normal control	−	92.17 ± 6.53 a	15.25 ± 1.35 c	1.84 ± 0.16 a	28.21 ± 2.27 d
Diabetic control	+++	284.75 ± 15.86 d	10.20 ± 0.90 a	4.23 ±0.40 c	16.51 ± 1.46 a
Diabetic + WSREt (100 mg/kg body weight)	+	136.05 ± 4.92 c	12.92 ± 1.14 bc	2.05 ± 0.15 ab	22.39 ± 1.09 b
Diabetic + WSREt (200 mg/kg body weight)	+	100.12 ± 9.67 c	14.60 ± 1.29 de	1.90 ± 0.18 ab	24.78 ± 2.19 c
Diabetic + WSLEt (100 mg/kg body weight)	+	132.98 ± 6.52 c	12.28 ± 0.64 b	1.80 ± 0.09 a	25.45 ± 1.85 c
Diabetic + WSLEt (200 mg/kg body weight)	+	106.19 ± 4.71 b	14.82 ± 1.35 de	1.85 ± 0.18 a	28.14 ± 2.16 d
Diabetic + glibenclamide	+	110.77 ± 2.75 b	13.76 ± 1.26 cd	2.15 ± 0.20 b	27.71 ± 1.45 d

Values are expressed as mean ± SD of six samples from each group. Values with the same letter within columns are not significantly different using Duncan’s Multiple Range Test at 5% level (p ≤ 0.05). WSREt: *W. somnifera* root extract, WSLEt: *W. somnifera* leaf extract, haemoglobin (Hb), glycosylated haemoglobin (HbA1C).

**Table 4.** Effects of *W. somnifera* (L.) extracts on serum lipid profile.

**Groups**	**TC (mg/dL)**	**TG (mg/dL)**	**PL (mg/dL)**	**HDL-c (mg/dL)**	**VLDL-c (mg/dL)**	**LDL–c (mg/dL)**
Normal control	94.62 ± 6.71 a	78.14 ± 6.92 a	86.22 ± 7.64 a	24.08 ± 1.82 c	15.63 ± 1.38 a	38.43 ± 7.04 a
Diabetic control	190.42 ± 16.20 c	114.82 ± 10.17 c	138.43 ± 12.26 b	16.82 ± 1.59 a	22.96 ± 2.03 c	75.03 ± 4.55 c
Diabetic + WSREt	102.46 ± 6.92 a	86.43 ± 7.65 ab	89.89 ± 7.96 a	22.31 ± 1.76 bc	17.29 ± 1.53 ab	47.83 ± 4.81 b
Diabetic + WSLEt	116.5 ± 8.20 b	90.86 ± 8.05 b	92.11 ± 6.69 a	20.46 ± 1.68 b	18.17 ± 1.61 b	52.23 ± 8.72 b
Diabetic + glibenclamide	100.44 ± 4.93 a	88.44 ± 6.42 b	87.66 ± 6.37 a	22.35 ± 1.84 bc	17.69 ± 1.28 b	48.41 ± 4.54 b

Values are expressed as mean ± SD of six samples from each group. Values with the same letter within colums are not significantly different using Duncan’s Multiple Range Test (DMRT) at 5% level (p ≤ 0.05). Both WSREt and WSLEt were treated at 200 mg/kg bw. WSREt: *W. somnifera* root extract, WSLEt: *W. somnifera* leaf extract, TC: total cholesterol, TG: triglycerides, PL: phospholipids, HDL-c: high density lipoprotein-bound cholesterol, VLDL-c: very low density lipoprotein-bound cholesterol, LDL-c: low density lipoprotein-bound cholesterol.

**Table 5.** Effects of *W. somnifera* (L.) extracts on liver and kidney lipids.

**Groups**	**Liver**			**Kidney**		
	**TC**	**TG**	**PL**	**TC**	**TG**	**PL**
Normal control	5.11 ± 0.32 a	7.27 ± 0.68 a	13.69 ± 1.28 a	6.22 ± 0.43a	4.88 ± 0.28 a	15.51 ± 1.48 a
Diabetic control	8.33 ± 0.86 d	10.89 ± 0.95 e	25.51 ± 1.86 d	9.97 ± 0.51e	9.12 ± 0.71 d	30.21 ± 2.53 e
Diabetic + WSREt	5.43 ± 0.71 c	7.69 ± 0.59 b	14.09 ± 0.98 b	6.82 ± 0.62 c	5.29 ± 0.47 b	16.29 ± 1.21 c
Diabetic + WSLEt	6.52 ± 0.65 cd	8.43 ± 0.48 d	16.81 ± 1.16 c	7.69 ± 0.53d	6.86 ± 0.36 c	20.34 ± 2.11 d
Diabetic + glibenclamide	5.29 ± 0.47 b	8.0 ± 0.92 c	14.21 ± 1.54 b	6.52 ± 0.61b	5.01 ± 0.53 b	16.05 ± 1.82 b

Values are expressed as mean ± SD of six samples from each group and represent mg/g wet tissue. Values with the same letter within colums are not significantly different using Duncan’s Multiple Range Test at 5% level (p ≤ 0.05). Both WSREt and WSLEt were treated at 200 mg/kg bw. WSREt: *W. somnifera* root extract, WSLEt: *W. somnifera* leaf extract, TC: total cholesterol, TG: triglycerides, PL: phospholipids.

**Table 6.** Effects of *W. somnifera* (L.) extracts on heart lipids.

**Groups**	**Heart**		

	**TC**	**TG**	**PL**
Normal control	3.13 ± 0.28 a	2.21 ± 0.21 a	8.16 ± 0.67 a
Diabetic control	6.65 ± 0.75 e	5.63 ± 0.52 e	16.23 ± 1.25 e
Diabetic + WSRET	3.49 ± 0.41 b	2.55 ± 0.26 bc	9.01 ± 0.61 c
Diabetic + WSLET	4.82 ± 0.28 d	3.64 ± 0.24 d	11.46 ± 0.53 d
Diabetic + glibenclamide	3.81 ± 0.31 c	2.43 ± 0.18 b	8.49 ± 0.49 b

Values are expressed as mean ± SD of six samples from each group. Values with the same letter within columns are not significantly different using Duncan’s Multiple Range Test at 5% level (p ≤ 0.05). Both WSREt and WSLEt were treated at 200 mg/kg body weight. WSREt: *W. somnifera* root extract, WSLEt: *W. somnifera* leaf extract, TC: total cholesterol, TG: triglycerides, PL: phospholipids.

**Table 7.** Effects of *W. somnifera* (L.) extracts on serum and tissue (liver, kidney and heart) proteins.

**Groups**	**Serum total protein (g/dL)**	**Albumin (g/dL)**	**Globulin (g/dL)**	**A:G ratio**	**Liver protein**	**Kidney protein**	**Heart protein**
Normal control	7.13 ± 1.76 b	4.87 ± 2.56 b	2.44 ± 0.18 a	2.04 ± 0.11 b	8.24 ± 2.38 b	7.62 ± 1.96 b	6.31 ± 1.76 b
Diabetic control	5.60 ± 0.53 a	3.21 ± 0.31 a	2.42 ± 0.21 a	1.34 ± 0.09 a	4.18 ± 0.39 a	5.26 ± 0.50 a	4.16 ± 0.39 a
Diabetic + WSREt	6.93 ± 0.56 b	4.71 ± 0.60 b	2.18 ± 0.12 a	2.16 ± 0.13 b	7.91 ± 0.56 b	7.44 ± 0.51 b	6.18 ± 0.80 b
Diabetic + WSLEt	7.08 ± 0.64 b	4.64 ± 1.60 b	2.35 ± 0.22a	1.98 ± 0.10 b	7.62 ± 0.59 b	6.81 ± 0.70 b	5.96 ± 0.99 b
Diabetic + glibenclamide	6.85 ± 0.53 b	4.60 ± 1.17 b	2.30 ± 0.19a	2.00 ± 0.12 b	8.01 ± 0.84 b	7.69 ± 0.72 b	5.88 ± 0.66 b

Values are expressed as mean ± SD of six samples from each group. Values with the same letter within columns are not significantly different using Duncan’s Multiple Range Test at 5% level (p ≤ 0.05). Both WSREt and WSLEt were treated at 200 mg/kg body weight. Tissue proteins are expressed as g/100g wet tissue. WSREt: *W. somnifera* root extract, WSLEt: *W. somnifera* leaf extract, A:G ratio: albumin/globulin.

**Table 8.** Effects of *W. somnifera* (L.) extracts on liver G6P and serum enzymes AST, ALT, ACP and ALP.

**Groups**	**G6P**	**AST**	**ALT**	**ACP**	**ALP**
Normal control	11.68 ± 1.19 a	92.86 ± 6.64 a	69.38 ± 7.11 a	5.88 ± 1.78 a	17.93 ± 1.92 a
Diabetic control	25.72 ± 1.68 d	168.23 ± 7.12 c	144.19 ± 6.96 d	9.23 ± 2.00 b	22.19 ± 3.00 b
Diabetic + WSREt	14.22 ± 0.97 c	92.45 ± 6.53 a	86.27 ± 5.96 b	6.56 ± 2.22 a	18.62 ± 1.02 a
Diabetic + WSLEt	13.37 ± 0.90 bc	102.11 ± 6.89 b	79.05 ± 5.34 b	6.32 ± 0.71 a	18.92 ± 2.11 a
Diabetic + glibenclamide	12.18 ± 1.65 ab	96.68 ± 5.01 ab	76.44 ± 4.62 ab	5.92 ± 0.61 a	18.21 ± 2.74 a

Values are expressed as mean ± SD of six samples from each group. Values with the same letter within columns are not significantly different using Duncan’s Multiple Range Test at 5% level (p ≤ 0.05). Both WSREt and WSLEt were treated at 200 mg/kg body weight. G6P is expressed as μ mole of pi liberated/min/mg protein and AST. ALT, ACP and ALP are expressed as IU/L. WSREt: *W. somnifera* (L.) root extract, WSLEt: *W. somnifera* (L.) leaf extract, G6P: glucose-6-phosphatase, AST: aspartate transaminase, ALT: alanine transaminase, ACP: acid phosphatase, ALP: alkaline phosphatase.

## References

[1] Akhtar FM, Ali MR (1984). Study of antidiabetic effect of a compound medicinal plant prescription in normal and diabetic rabbits. J. Pak. Med. Assoc.

[2] Brinker F (1998). Herb contraindications and drug interactions.

[3] Pepato MT, Baviera AM, Vendramini RC, Perez MPMS, Ketelhut IC, Brunetti ILJ (2003). *Cissus sicyoides* (princess vine) in the longterm treatment of streptozotocin-diabetic rats. Biotechnol. Appl. Biochem.

[4] Budhiraja RD, Sudhir S (1987). Review of biological activity of withanolides. J. Sci. Ind. Res.

[5] Chopra RN (1994). Glossary of Indian medicinal plants.

[6] Glotter E, Kirson I, Abraham A, Lavie D (1973). Constituents of *Withania somnifera* (L.) Dunal. XII. The withanolides of chemotype III. Tetrahedron.

[7] Devi PU, Sharada AC, Solomon FE (1993). Anti-tumor and radiosensitizing effects of *Withania somnifera* (Ashwagandha) on a transplantable mouse tumor sarcoma 180. Indian J. Exp. Biol.

[8] Nittala SS, Lavie S (1988). Chemistry and genetics of withanolides in *Withania somnifera* hybrids. Phytochemistry.

[9] Kandil FE, Elsayeh NH, Abou-Douh AM, Ishak MS, Mabry TJ (1994). Flavonol glycosides and phenolics from *Withania somnifera*. Phytochemistry.

[10] Bhattacharya SK, Satyam SK, Chakrabarti A (1997). Effect of Trasina an Ayurvedic herbal formulation, on pancreatic islet superoxide dismutase activity in hyperglycaemic rats. Indian J. Exp. Biol.

[11] Tripathi AK, Shukla YN, Sushilkumar T (1996). Ashwagandha *Withania somnifera* (L.) Dunal (Solanaceae): A status report. J. Med. Arom. Plant Sci.

[12] Andallu B, Radhika B, Dawar R (2000). Hypoglycaemic, diuretic and hypocholesterolemic effects of winter cherry *Withania somnifera* (L.) Dunal root. Indian J. Exp. Biol.

[13] Anwer T, Sharma M, Pillai KK, Iqbal M (2008). Effect of *Withania somnifera* on insulin sensitivity in non insulin dependent diabetes mellitus rats. Basic Clin. Pharmacol. Toxicol.

[14] Manach C, Scalbert A, Morand C, Remesy C, Jimenez L (2004). Polyphenols: food sources and bioavailability. Am. J. Clin. Nutr.

[15] Sharma B, Viswanath G, Salunke R, Roy P (2008). Effects of flavonoid-rich extract from seeds of *Eugenia jambolana* (L.) on carbohydrate and lipid metabolism in diabetic mice. Food Chem.

[16] Sharma SB, Nasir A, Prabhu KM, Murthy PS, Dev G (2003). Hypoglycaemic and hypolipidemic effect of ethanolic extract of seeds *Eugenia jambolana* in alloxan-induced diabetic rats. J. Ethnopharmacol.

[17] Ju JB, Kim JS, Choi CW, Lee HK, Oh TK, Kim SC (2008). Comparison between ethanolic and aqueous extracts from Chinese juniper berries for hypoglycaemic and hypolipidemic effects in alloxan-induced diabetic rats. J. Ethnopharmacol.

[18] Panten U, Schwanstecher M, Schwanstecher C (1996). Sulfonylurea receptors and mechanism of sulfonylurea action. Exp. Clin. Endocrinol. Diabetes.

[19] Luzi L, Pozza G (1997). Glibenclamide: an old drug with a novel mechanism of action. Acta Diabetol.

[20] Guo L, Hu WR, Lian JH, Ji W, Deng T, Qian M (2006). Anti-hyperlipidemic properties of CM 108 (a flavone derivative) in vitro and in vivo. Eur. J Pharmacol.

[21] Babu PS, Prabuseenivasan S, Ignacimuthu S (2007). Cinnamaldehyde-A potential antidibetic agent. Phytomedicine.

[22] Al-Yassin D, Ibrahim K (1981). A minor haemoglobin fraction and the level of fasting blood glucose. J. Fac. Med. Bagh.

[23] Punitha R, Manoharan S (2006). Antihyperglycemic and antilipidperoxidative effects of *Pongamia pinnata* (Linn.) *Pierre* flowers in alloxan induced diabetic rats. J. Ethnopharmacol.

[24] Huang X, Vagg A, Hanson M, Weng J, Laurila E, Group L (2000). Impaired insulin stimulated expression of the glycogen synthase gene in skeletal muscle of type 2 diabetic patients is acquired rather that inherited. J. Clin. Endocrinol. Metab.

[25] Leite ACR, Aráujo TG, Carvalho BM, Silva NH, Lima VLM, Maia MBS (2007). *Parkinsonia aculeata* aqueous extract fraction: Biochemical studies in alloxan-induced diabetic rats. J. Ethnopharmacol.

[26] Bhattacharya SK, Satyan KS, Ghosal S (1997). Antioxidant activity of glycowithanolides from *Withania somnifera*. Indian J. Exp. Biol.

[27] Sivajothi V, Dey A, Jayakar B, Rajkapoor B (2007). Antihyperglycemic property of *Tragia cannabina* in streptozotocin-induced diabetic rats. J. Med. Food.

[28] Pathak A, Dhawan D (1998). Effect of lithium on the levels of blood urea and creatinine in diabetic rats. Med. Sci. Res.

[29] Anbalagan K, Sadique J (1981). Influence of an Indian Medicine (Ashwagandha) on acute phase reactants in inflammation. Indian J. Exp. Biol.

[30] Gokce G, Haznedaroglu MZ (2008). Evaluation of antidiabetic, antioxidant and vasoprotective effects of *Posidonia oceanica* extract. J. Ethnopharmacol.

[31] Prince PSM, Menon VP (2000). Hypoglycaemic and other related actions of *Tinospora cardifolia* roots in alloxan – induced diabetic rats. J. Ethnopharmacol.

[32] Bhavapriya V, Kalpana S, Govindasamy S, Apparantham T (2001). Biochemical studies on hypoglycaemic effect of Aavirai kudineer: a herbal formulation in alloxan diabetic rats. Indian J. Exp. Biol.

[33] Batran SAS, El-Gengaihi SE, Shabrawy OA (2006). Some toxicological studies of *Momordica charantia* L. on albino rats in normal and alloxan diabetic rats. J. Ethnopharmacol.

[34] Prince PSM, Menon VP, Pari L (1997). Effect of *Syzigium cumini* extracts on hepatic hexokinase and glucose-6-phosphatase in experimental diabetes. Phytother. Res.

[35] Quettier-Deleu C, Gressier B, Vasseur J, Dine T, Brunet C, Luyckx M, Cazin M, Cazin JC, Bailleul F, Trotin F (2000). Phenolic compounds and antioxidant activities of buckweat hulls and flour. J. Ethnopharmacol.

[36] Prince PSM, Menon VP, Pari L (1998). Hypoglycaemic activity of *Syzigium cumini* seeds: effect on lipid peroxidation in alloxan diabetic rats. J. Ethnopharmacol.

[37] Folch J, Lees M, Slone Stanley GHS (1957). A simple method for the isolation and purification of total lipids from animal tissues. J. Biol. Chem.

[38] Varley H (2003). Practical Clinical Biochemistry.

[39] Sasaki T, Matsy S, Sonae A (1972). Effect of acetic acid concentration on the colour reaction in the O-toluidine boric acid method for blood glucose estimation. Rinsho Kagaku.

[40] Dacie JV, Lewies SM (1977). Practical Haematology.

[41] Bannon P (1982). Effect of pH on the estimation of the liable fraction of glycosylated haemoglobin. Clin. Chem.

[42] Carroll NV, Longly RW, Joseph HR (1956). Determination of glycogen in liver and muscle by use of anthrone reagent. J. Biol. Chem.

[43] Zlatkis A, Zak B, Boyle AJ (1953). A method for the direct determination of serum cholesterol. J. Lab. Clin. Med.

[44] Foster CS, Dunn O (1973). Stable reagents for determination of serum triglycerides by a colorimetric hantzsch condensation method. Clin. Chem.

[45] Zilversmit DB, Davies AK (1950). Micro determination of plasma phospholipids by TCA precipitation method. J. Lab. Clin. Med.

[46] Burstein M, Scholnick HR, Morgin R (1970). Rapid method for the isolation of lipoprotein from human serum by precipitation with polyanion. J. Lipid Res.

[47] Lowry OH, Roesborough MJ, Farr AL, Randall RJ (1951). Protein measurement with Folin’s-phenol reagent. J. Biol. Chem.

[48] Reinhold JG, Reiner M (1952). Standard methods in clinical chemistry.

[49] Koide H, Oda T (1959). Pathological occurrence of glucose-6-phosphatase in serum and liver diseases. Clin. Chim. Acta.

[50] Reitman S, Frankel SA (1957). Colorimetric method for the determination of serum glutamate oxaloacetate and glutamate pyruvate transaminases. Am. J. Clin. Pathol.

[51] King J (1959). Determination of serum alkaline and acid phosphatase. Practical Clinical Enzymology.

